# Effect of Wood Dust Fibre Treatments Reinforcement on the Properties of Recycled Polypropylene Composite (r-WoPPC) Filament for Fused Deposition Modelling (FDM)

**DOI:** 10.3390/ma16020479

**Published:** 2023-01-04

**Authors:** Z. A. S. Nafis, M. Nuzaimah, S. I. Abdul Kudus, Y. Yusuf, R. A. Ilyas, V. F. Knight, M. N. F. Norrrahim

**Affiliations:** 1 Fakulti Kejuruteraan Mekanikal, Universiti Teknikal Malaysia Melaka, Hang Tuah Jaya, Durian Tunggal 76100, Melaka, Malaysia; 2 Fakulti Teknologi Kejuruteraan Mekanikal dan Pembuatan, Universiti Teknikal Malaysia Melaka, Hang Tuah Jaya, Durian Tunggal 76100, Melaka, Malaysia; 3 Faculty of Chemical and Energy Engineering, Universiti Teknologi Malaysia, Johor Bahru 81310, Johor, Malaysia; 4 Centre for Advanced Composite Materials, Universiti Teknologi Malaysia, Johor Bahru 81310, Johor, Malaysia; 5 Institute of Tropical Forestry and Forest Products, Universiti Putra Malaysia, Serdang 43400, Selangor, Malaysia; 6 Centre of Excellence for Biomass Utilization, Universiti Malaysia Perlis, Arau 02600, Perlis, Malaysia; 7 Research Centre for Chemical Defence, Universiti Pertahanan Nasional Malaysia, Kem Perdana Sungai Besi, Kuala Lumpur 57000, Malaysia

**Keywords:** wood dust fibre, fibre treatment, polymer composites, mechanical properties, thermal properties, cellulose, FDM

## Abstract

The efficacy of wood dust fibre treatment on the property of wood dust reinforced recycled polypropylene composite (r-WoPPC) filament was investigated. The wood dust fibre was treated using alkali, silane, and NaOH-silane. The treated wood fibre was incorporated with r-PP using a twin-screw extruder to produce filament. The silane treatment on wood dust fibre enhances interfacial bonding between wood fibre and recycled PP; hence, a filament has the highest wire pull strength, which is 35.2% higher compared to untreated and alkaline-treated wood dust filament. It is because silanol in silane forms a siloxane bond that acts as a coupling agent that improves interfacial bonding between wood dust fibre and recycled PP. The SEM micrograph of the fracture structure reveals that treated silane has strong interfacial bonding between wood dust fibre and recycled PP, having minimal void, gap, and good fibre adhesion. The water absorption test results indicate that filament with treated wood dust absorbs less water than filament with untreated wood because the treatment minimizes the gap between wood fibres and recycled PP. The FTIR analysis identified the presence of silane on the wood dust surface for silane-treated wood dust. The DSC studies suggest that the temperature range 167–170 °C be used in the extrusion machine to produce r-WoPPC filament. As a result, r-WoPPc filaments containing silane-treated wood dust have better mechanical properties and have a greater potential for usage in FDM applications.

## 1. Introduction

Materials research and development, notably in the composite industry, is being pushed forward by a growing demand for high-tech material products [1]. Due to advances in fabric science, it is now possible to use natural fibres in stylish applications because of their prominence in the field. Materials that are both more cost-effective and ecologically friendly are now being developed because of recent expansions in research into current, higher performing front-property industrial material sources (FPIM) [2]. Concerns regarding the overexploitation of non-traditional sources of natural fibres are on the rise as people become more environmentally conscious and turn to recycled and sustainable materials.

Local governments are struggling to manage a rising amount of solid waste, not only because of the volume, but also because of the variety of waste sources derived from natural or synthetic resources that need a combination of treatment procedures. Synthetic polymers and plastics are extensively employed owing to their cheap cost, outstanding physical, chemical, and mechanical properties, and processing flexibility. Between 1950 and 2015, around 8300 metric tonnes of synthetic polymers were produced worldwide. Natural trash was easily disposed of due to its quantity, accessibility, and cheap cost [3,4].

Meanwhile, because it has a substantial influence on environmental biodiversity, the utilisation of bio-based waste, such as natural fibre waste, has been a vital component of furthering sustainable development projects. Utilizing plentiful bio-resources has facilitated the development of a range of materials for several applications, the most significant of which is the fabrication of ecologically acceptable bio-composite materials [5,6,7,8,9]. Additionally, plastic waste from businesses and households was extensively used in the manufacture of bio-composite materials, which was one of the primary efforts to embrace the 3R concept (reduce, reuse, and recycle) and promote sustainable development, but, similar to PP, they have low strength and modulus, hence they cannot take higher loads during applications. Reinforcement of polymer with fibre can improve the strength of the composites [10,11,12].

Natural fibres are generated from a range of plant, animal, and mineral sources [13]. There is an abundance of fibrous plants in both agricultural and tropical areas [14,15,16]. Most plant fibres are composed of cellulose, whereas most animal fibres are composed of protein [17,18,19,20]. The primary and secondary sources of plant fibres are distinguished [21]. Although secondary sources are fibres produced as by-products of other secondary products obtained from manufacturing processes, primary sources are fibres produced as by-products of other primary items (e.g., food, feedstock, and fuel) intended for industrial use [22,23,24,25]. Table 1 shows mechanical and physical properties of natural fibres.

Wood dust, which is mostly composed of cellulose, hemicellulose, and lignin, is inexpensive and simple to make. Due to the strength and flexibility of wood fibres and the stiffness of lignin, wood powder is an excellent filler for matrix. Additionally, wood powder can be used to cure scrap wood, such as waste furniture. In the same manner, secondary timber may be utilised without generating environmental problems. Wood dust is basically the residue of small particles found in the furniture industry, pulp and paper industry, can cause pile shapes, and mostly burns, resulting in environmental pollution. Polypropylene (PP) is a common recyclable engineering thermoplastic [29,30]. Textile and other sectors use matrix composite materials manufactured from recycled PP fibres. Polypropylene has a low modulus. Wood powder may be injected into polypropylene to reinforce it. Polypropylene can bind wood powder. Unlike other composite materials, PP-based wood powder composites may be recycled after use.

Plastic waste from petroleum-based polymers, such as polypropylene, polyethylene terephthalate, high-density polyethylene (HDPE), and polyethylene (PE), are becoming a critical issue because they cannot be degraded [31]. PP and PET are not environmentally friendly but are eco-friendly substances that allow them to be recycled after use, turning waste plastics such as PP and PET into valuable resources that are recycled for a variety of uses, including the sustainable manufacturing of sustainable polymer matrix composite materials [32].

For example, alkaline treatment fibres are often treated with NaOH because it is simple, affordable, and effective in enhancing filler-matrix adhesion [33]. NaOH affects the surface of the fibre by mechanically removing it, increasing surface roughness and, hence, matrix coverage. In addition, chemical treatment, namely silane treatment as a coupling agent to increase the wettability of natural fibres by polymer matrix and promote interfacial bonding, is another treatment [3,34]. To assess the characteristics of wood dust fibre composites, a thermal test is necessary. Table 2 shows a list of findings based on performance treatment used with different fibre composites.

Materials such as wood dust fibre polymer matrix recycled polypropylene (PP) will be examined along with their thermal properties such as TGA and DSC. A thermogravimetric analysis (TGA) analyser was used to evaluate the thermal stability of wood dust fibres in correlation to weight loss as a result of if the temperature was increased. TGA was performed using TA instrument’s equipment with filament samples in standard ASTM D3850.

The purpose of this study is to identify the effect of different treatments on wood dust fibre. This study tested three different wood dust treatments: NaOH, silane, and NaOH-silane. Processed treated wood dust was subjected to FTIR analysis. The filaments were produced using a twin-screw extruder and subjected to water absorption, tensile, morphological analysis, TGA, and DSC tests. It stems from a basic understanding of how the three treatments affect the properties of r-WoPPC filaments.

## 2. Materials and Methods

### 2.1. Materials

**Figure 1:**
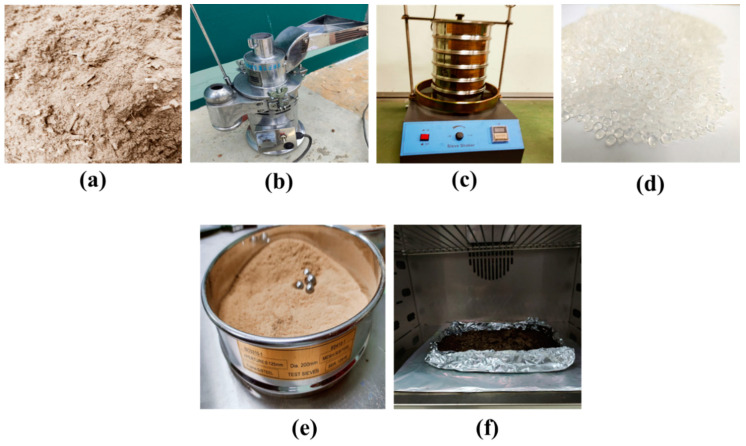
(**a**) Raw wood dust from workshop, (**b**) grinder machine for grinding into dust, (**c**) industrial sieve for obtaining a result of 125 µm, (**d**) recycled PP, (**e**) 125 µm wood dust inside sieve, (**f**) untreated wood dust inside oven.

The matrix and reinforcing materials engaged in the present research work are thermoplastic and wood-based dust fillers, respectively. The raw wood particles are obtained from a local furniture factory in Alor Gajah, Melaka, and are made up of pine grove and tongue wood. The wood waste is ground into a powder using a portable grinder at the FTKMP composite lab at the University Teknikal Malaysia Melaka, and then filtered using an industrial server, as shown in Figure 1b.

**Figure 2:**
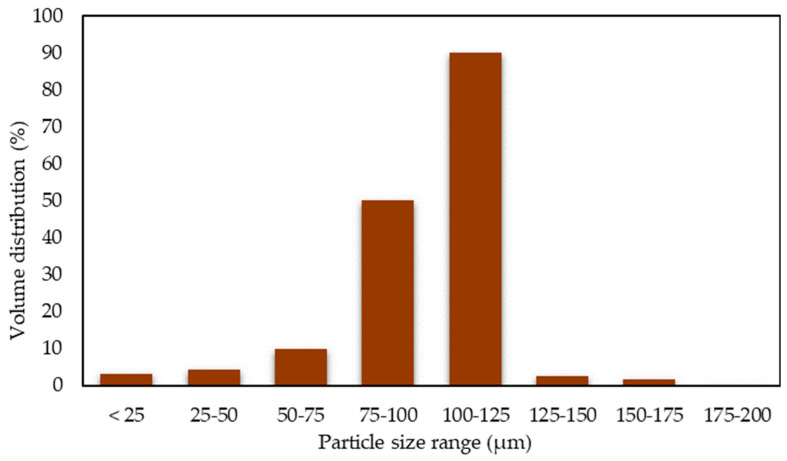
Diagram of wood dust particle size.

The sieve mesh sizes that were used ranged from 300 µm to 200 µm and, finally, 125 µm [39]. The finished product is a 125 µm mesh made of wood powder [35], as shown in Figure 1e. The particle size analyser equipment was utilised in the dry measurement mode, and a random 150 g sample of wood dust was obtained to evaluate the size content of the wood dust used, and Figure 2 tabulates the data resulting from the particle size analyser. This size was chosen wisely related to the review according to Nasereddin et al. [41] and Das et al. [42]. Cleaning wood filler in a mixture of dirt particles, grease, and tiny metal fragments is a particularly difficult process. The sieved particles are washed several times in distilled water to remove dust and other pollutants before being dried in a hot air oven at 60 °C for 24 h on a portion of an untreated wood dust sample. A plastic factory in Ayer Keroh, Melaka, provided recycled PP granules measuring 2 mm in diameter. The transparent milky white recycled PP used in this study is seen in Figure 1d.

Regarding the bar chart from Figure 2, particle size analysis is recorded by the machine, Malvern Mastersizer 3000 Aero S dry powder disperser. The sample dispersion is achieved by accelerating the dry powder particles through a venturi using compressed air and used on using mesh size of <125 μm with high volume distribution instead of a different mesh size, stating that with a mesh size of 125–200 μm, there is not more than 1% volume distribution. The total amount tested from a 200 g sieve of wood dust with a mesh size of 100–125 μm results in 90% detection during the analysis, The rest of the analysis at mesh size 75–100 μm is 50%, 50–75 μm is 10%, 25–50 μm is 5%, and a mesh size less than <25 is only 3% that can be detected.

### 2.2. Methods

#### 2.2.1. Alkaline Treatment of Wood Dust Fibre

Wood dust fibre treatment for alkaline treatment uses a sodium hydroxide solution. The reason for selecting the alkaline treatment process is the direct influence of the hydroxyl group on cellulosic fibrils, the extraction of lignin and hemicellulose compounds, and ease of processing [39]. The wood dust was soaked in NaOH solution with 6% concentration at room temperature for 2 h [41,42]. After that, the wood was washed several times thoroughly with running distilled water and dried in an oven at a temperature of 60 °C for 24 h [35]. The alkaline treatment is displayed in Figure 4b. The chemical reaction for NaOH displayed in Figure 5a.

#### 2.2.2. Silane Treatment of Wood Dust Fibre

**Figure 3:**
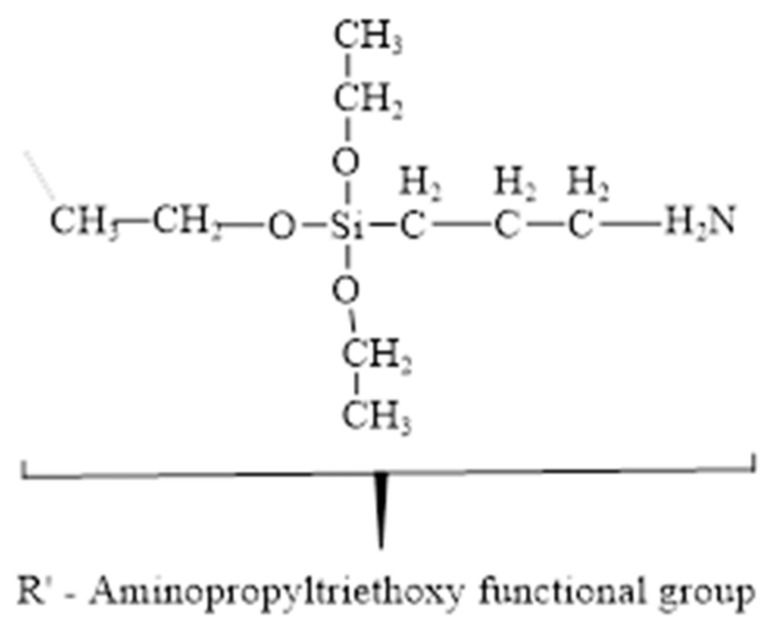
Chemical structure of Amino-silane (C_9_H_23_NO_3_Si).

**Figure 4:**
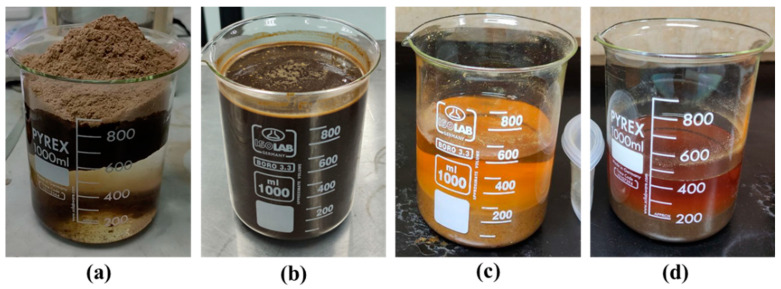
(**a**) Untreated wood wash, (**b**) NaOH treatment, (**c**) NaOH-silane treatment, and (**d**) Silane treatment.

**Figure 5:**
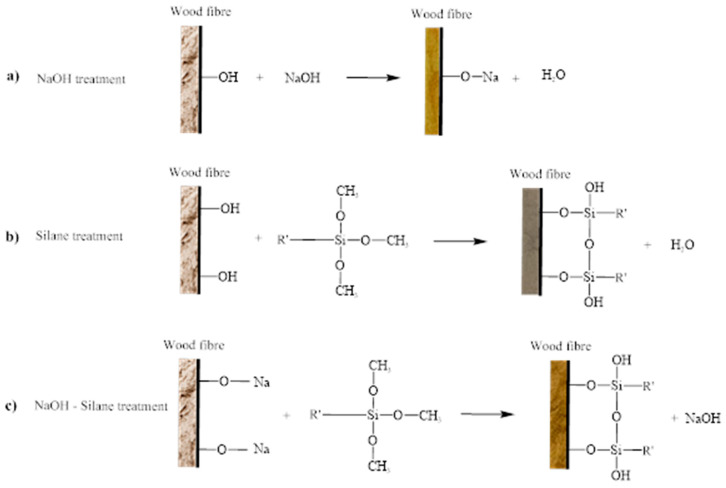
Chemical reactions for (**a**) NaOH treatment (**b**) silane treatment, and (**c**) NaOH-silane treatment.

Among these several methods, silane coupling agents have been commonly applied as efficient coupling agents in a variety of industrial sectors due to their simplicity of application, durability, and diversity, as well as low cost, in order to enhance the fibre-matrix adhesion strength [43]. The 2% silane solution was prepared by dissolving APS (3-Aminopropyltriethoxysilane) in a methanol solution (70% methanol and 30% distilled water). The methanol solution pH was adjusted to 3.5 using citric acid. The silane solution was agitated for 30 min [44]. Then, the wood dust fibre was soaked in silane solution for 3 h and dried in an oven at 60 °C for 72 h. The silane treatment is displayed in Figure 4c. Figure 3 shows a representation of the chemical structure for the silane functional group Amino-silane (C9H23NO3Si) [45,46]. The chemical reaction of the treated silane (Si) is displayed in Figure 5b.

#### 2.2.3. NaOH-Silane Treatment of Wood Dust Fibre

Another treatment method is to combine alkaline and silane treatments. The preparation of this sample begins with an alkaline treatment using NaOH. Once the sample of NaOH has dried completely, a silane treatment is applied by soaking it in a silane solution for 3 h and drying for 3 days, referring to the procedure conducted by Asim et al. (2016) [3], and renamed NaOH-silane treatment. Figure 4d shows the NaOH-silane treatment sample. The chemical reaction of the treated silane fibres is displayed in Figure 5c, which begins with Na + OH for the NaOH reaction and connected with the silane (Si) treatment reaction, then completed with the NaOH-silane reaction, following the structure by Raharjo et al. [44,47].

### 2.3. Filament Preparation

#### 2.3.1. Composite Mixture

The filament recycled PP and wood dust fibres were made utilizing the rule of the com-bination formula shown in Table 3. The weight of elements has been assessed to determine the composition of composites using Equation (1),

(1) Weight of component (g)=Weight % of fibre loading(g)×Weight of composites(g) 

As this composite, which is made of wood and recycled polypropylene (PP), has absolute and complete bonding, it is given the name wood dust reinforced recycled polypropylene (r-WoPPC) composite FDM filament.

#### 2.3.2. r-WoPPC Filament Extrusion

**Figure 6:**
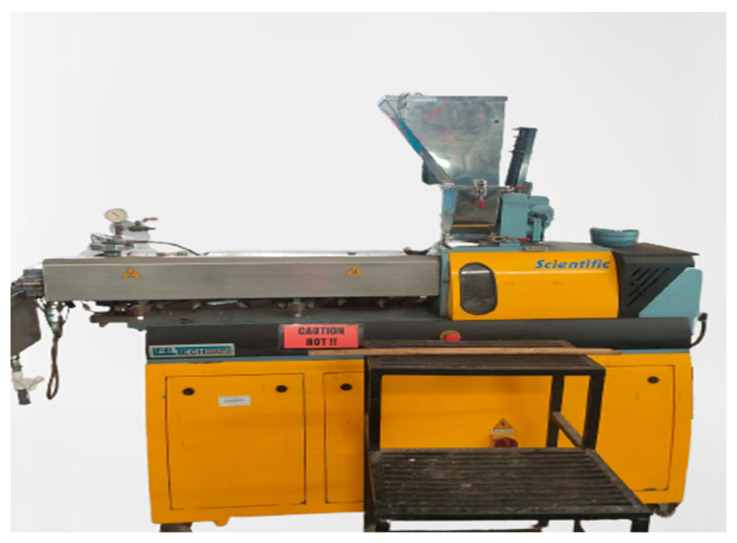
Lab Tech Twin Screw Extruder.

The extruder used was the Lab Tech Twin Screw Extruder 26 mm (Swedish), as shown in Figure 6, located at the Fibre and Bio composite Development Centre (FIDEC)—MTIB Malaysia, to produce r-WoPPC filament with different parameters, as shown in Table 3. Fixed parameters were a composite compound feeding screw of 42 rpm and a constant target filament diameter of 1.5–1.79 mm. Table 4 shows barrel temperature as a reference to extrude FDM filament.

#### 2.3.3. Density Calculation

The test method for determining the filament’s density was conducted in accordance with ASTM D792-91. Filament with a diameter of 1.75 mm ± 0.55 mm and a length of 90 mm were cut, and their densities were estimated using Equation (2):

(2) $$Density\;\left(\rho \right)=\frac{Mass\;of\;filament\;composite\;\left(g\right)}{Volume\;of\;filament\;composites\;m^{3}}$$

### 2.4. Testing

#### 2.4.1. Water Absorption Test of the r-WoPPC Filaments

The water absorbtion characteristics of composites were measured in accordance with the 2018 ASTM D570-98 standard. For each set of fibres, five composite samples measuring 25.4 mm × 1.75 ± 0.55 mm were created. Specimens were dried in an oven at 110 °C for 24 h before being cooled to room temperature for 24 h. After measuring their weight, they were submerged for 14 days at room temperature in distilled water. The wet surface was wiped dry, and the specimens were reweighed. The water uptake of composites was computed using Equation (3):

(3) $$Water\;Absorption\;\left(\%\right)=\frac{W_{1}-W_{0}}{W_{0}}\times 100\%$$

where $W_{0}$ (g) is the weight of composites after drying and, $W_{1}$ (g) is the weight after water immersion.

#### 2.4.2. Wire Pull Test

**Figure 7:**
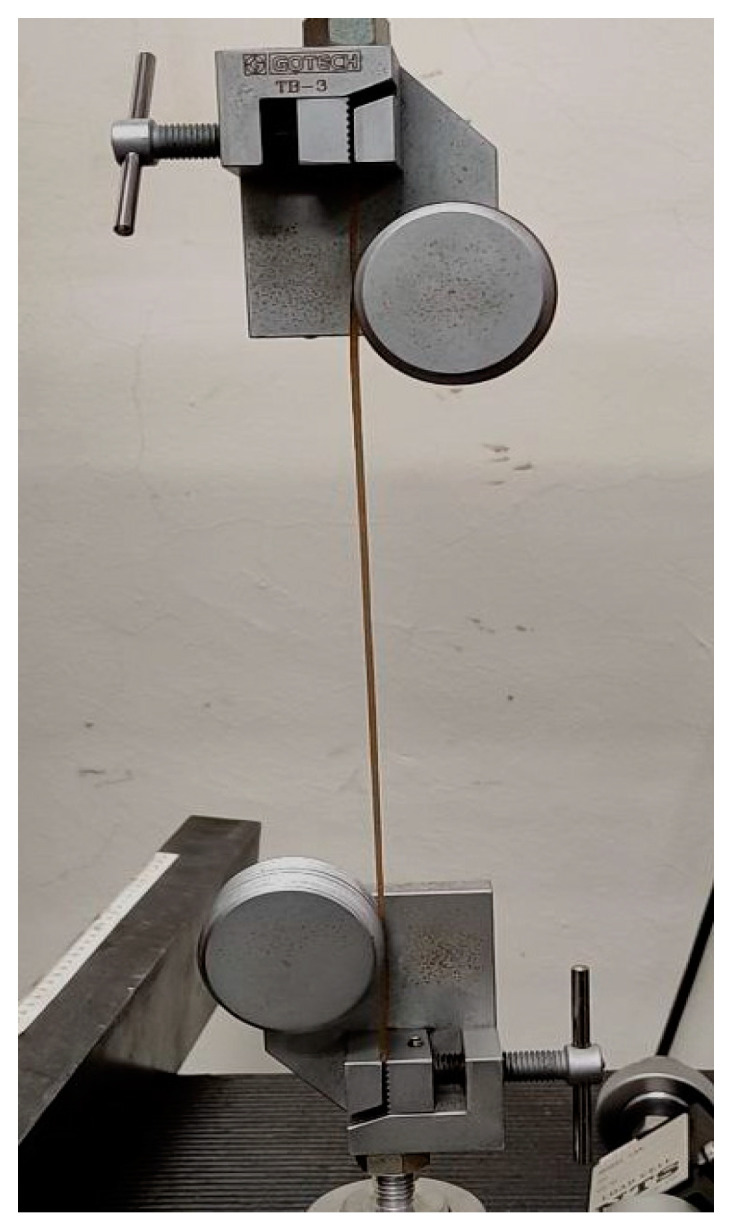
Wire pull test.

A wire pull test in accordance with ISO 3341 was carried out to determine the mechanical strength of the filament. Ferreira et al. [48] previously conducted this test for polyethylene terephthalate glycol/carbon fibre filament composites. Figure 7 shows the test setup on an AI-7000-LAU GoTech Servo Control System Universal Testing Machine with a 10 kN load cell at 200 mm/min speed. The test was repeated 5 times for each filament. The test setup must be properly prepared since r-WoPPC is more brittle than r-PP because it is mixed with wood fibre, thus the winding of the filament around the bollard must be performed carefully to avoid fractures during the test [49].

#### 2.4.3. Morphological Observations

Scanning electron microscope (SEM) morphological analyses were carried out on the fractured surface of the wire pull-out test sample for this study. These 4 types of treatment were examined with the same fibre loading of 3.0% point. The total five samples collected from the wire pull-out specimen were also tested. To improve resolution, platinum was applied to the samples, which has a high electrical conductivity. The micrograph was attained owing to the use of Zeiss Evo 18 Research, (Jena, Germany) with an acceleration voltage of 10 kV.

The measurement of the diameter for filament was by using a portable microscope up to 1600 magnification, which has many functions, including a measurement size from 0.001 mm to 50.00 mm. This method was used for 5 sample filaments, which are r-PP, untreated, and treated wood.

#### 2.4.4. The Fourier Transform Infrared Spectrometry (FTIR)

Fourier transform infrared spectrometry (FTIR) was performed on four samples of wood dust using a Thermo Fisher Scientific-The Nicolet iS10 FTIR Spectrometer to determine the functional group for each treatment: untreated, treated NaOH, treated silane, and treated NaOH-silane. All spectra were taken in 4000 cm^−1^ to 400 cm^−1^ range. The resolution is measured at a value of 0.5 cm^−1^ with 50 scans.

#### 2.4.5. Thermogravimetric Analysis

A thermogravimetric analysis (TGA) analyser was used to evaluate the thermal stability of wood dust fibres in correlation to weight loss as a result of if the temperature was increased. TGA was performed utilizing samples of filament granules in accordance with ASTM D3850, using a weight of 20 mg. Temperature rate of 30 to 800 °C using a flow rate of gas is 20 °C/min. TGA was obtained using Mettler-Toledo brand Thermogravimetric Analyzer TGA 2—Thermogravimetric Analyzer with small furnace (SF) specification; temperatures ranged up to 1100 °C and crucible volume up to 100 µL. Usually, a dynamic nitrogen atmosphere is present during operation and a 10 °C/min heating rate.

#### 2.4.6. Differential Scanning Calorimetry

A differential scanning calorimetry (DSC) investigation at inert atmosphere was performed to find more about the heat transfer in the sample. Crystal transition temperature, glass transition temperature, melting point, solvent evaporation, and residual reactivity all seem to be examples of transitions. DSC was performed using instrument equipment with filament granule samples in standard ASTM D3418. DSC analysis was performed with a heating rate of 20 °C/min, from 30 °C to 800 °C. Differential scanning calorimetry (DSC) was obtained using Mettler-Toledo brand DSC Q20 V24.11 Build 124 at FTKMP UTeM.

## 3. Results and Discussion

### 3.1. Physical Properties of r-WoPPC Filaments

#### Filament Size and Colour

**Figure 8:**
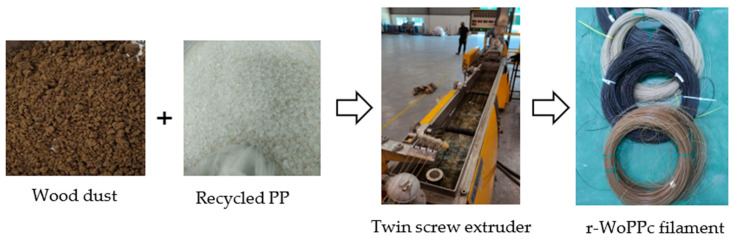
Process fabrication of r-WoPPc filament.

**Figure 9:**
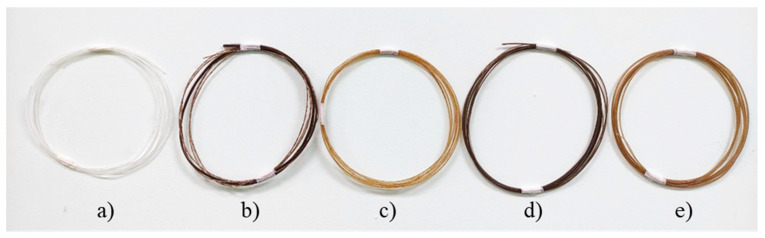
Filament r-WoPPC (**a**) r-PP, (**b**) 3/UT, (**c**) 3/NO, (**d**) 3/SI, and (**e**) 3/NS.

**Figure 10:**
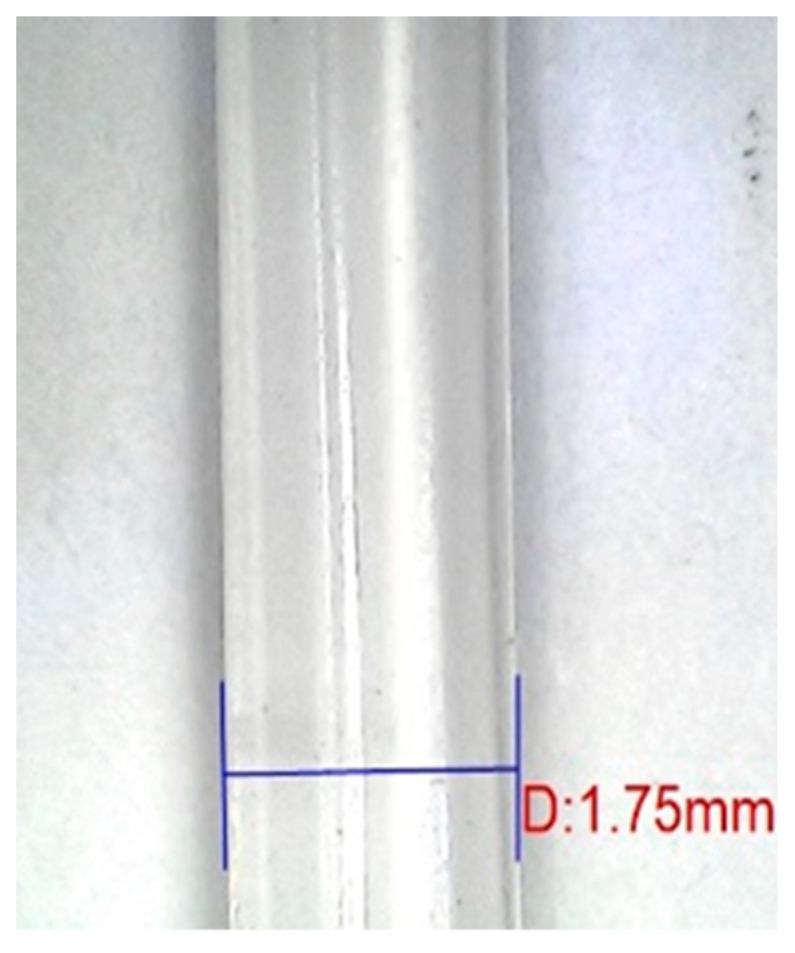
Filament diameter, 1.75 mm.

The fabrication of r-WoPPC filament was achieved using LabTech’s twin screw extrusion. Figure 8 shows filament development using the twin screw extruder. Figure 9 displays each filament that was recycled: PP, 3/UT, 3/NO, 3/SI, and 3/NS r-WoPPC filament that was successfully extruded. All the filaments were extruded at nozzle temperatures ranging from 160 °C to 200 °C with filament pulley speeds ranging from 10 rpm to 12 rpm. The nozzle temperature and pulley speed were adjusted to obtain a filament diameter of 1.75 mm, as shown on the label dimensions in Figure 10. According to the observations, 1.75 mm filament could be produced at nozzle extrusion temperatures ranging from 180 to 190 °C, as shown in Table 5, and pulley speed ranging from 7.1 rpm to 10 rpm, as tabulated in Table 6.

**Figure 11:**
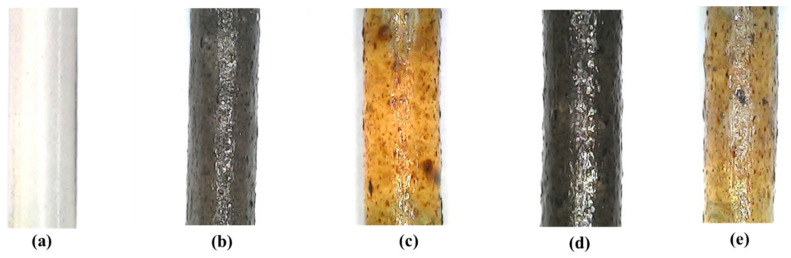
Microscopic image of filament (**a**) r-PP, (**b**) 3/UT, (**c**) 3/NO (**d**) 3/SI, and (**e**) 3/NS.

Based on the filament extrusion timing, as shown in Table 6, the fastest filament extrusion is r-PP filament, which is 6 s/min with a pulley speed of 10 rpm. This is because r-PP is purely matrix without any filler, which could cause friction and delay the extraction time. The extrusion time increased to 13 s/m when wood dust fibre was incorporated into the recycled PP. Since the treatment had an effect on the extrusion time, the 3/UT filament had a faster extrusion time than the other treated filaments, as reported by Siddique et al. [50]. Figure 11 shows a microscopic image of r-PP and r-WoPPC filaments. The filament exhibits a distinct colour for each r-WoPPc filament, which is dark brown for 3/UT, light orange for 3/NO, light black for 3/SI, and light brown for 3/NS.

Silane-created layers on the fibre surface encouraged an increase in fibre density; thus, we can see filament densities in Table 7, showing that densities of 3/SI and 3/NS have the highest density values of 0.99 g/cm^3^ and 1.01 g/cm^3^, respectively. Silane layer that developed on the wood surface enhanced adhesion with recycled PP, hence giving better filament strength [36]. Furthermore, the r-PP filament that is 0.85 g/cm^3^, which is as a neat filament, does not contain any mixture with fibre, making it among the lowest compared to others where it also shows a close resemblance to the commercial value of PP [51]. The filament with the lowest density is 3/UT with 0.82 g/cm^3^. This might be due to untreated fibre that contains cellulose, hemicellulose, pectin, and lignin. These fibre compositions result in the lowest density among other treated filament composites [39]. The density of the filament is lower due to the presence of voids and voids in the sample. Air trapping during extrusion and moisture absorption during storage are the main causes of formation. The presence of voids and gaps resulted in weak interfacial adhesion between the fibre and the matrix, resulting in poor strength [49,52].

### 3.2. Water Absorption Test

**Figure 12:**
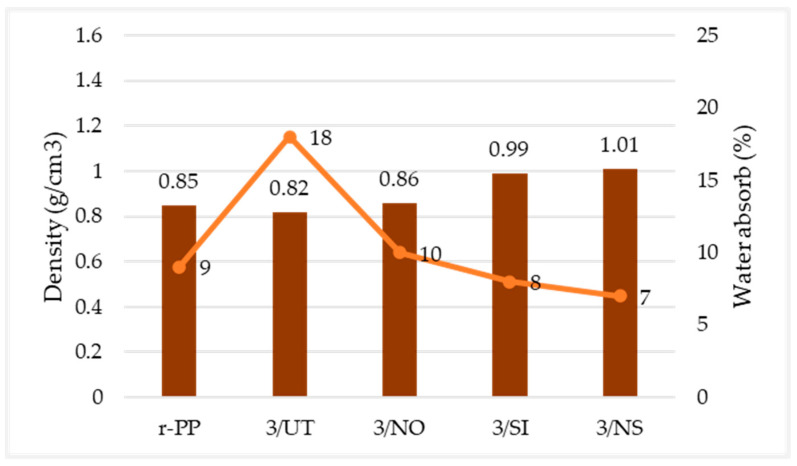
Graph of water absorption test—density of filament.

The percentage of water absorption test was calculated by weighing the wet and dry specimens and comparing the differences, as in Equation (3) [53]. Based on Figure 12, the water absorption of r-PP filament was lower, which is only 9%, because r-PP is pure polypropylene, which has hydrophobic properties that resist water; therefore, it absorbs minimal water and has a low moisture content [54]. In contrast, filament made of untreated wood fibre (3/UT) has the highest percentage of water absorption of 18%, because wood is a lignocellulose material that can absorb more moisture than pure polypropylene because it contains polar group-hydroxyl. As a result, composites containing wood powder absorb water better than those made of pure polypropylene [54,55]. Additionally, untreated wood fibres have natural oils, wax, pectin, and lignin content that prevent the wood fibre from adhering to the PP matrix effectively, resulting in more voids that cause the filament to absorb more water [53].

Meanwhile, silane treatment on the wood fibre resulted better adhesion between wood fibre and recycled PP. The adhesion was enhanced due to the presence of water in the silane solution that makes the aminopropyltriethoxy in silane produce silanol. Multiple silanol react with each other to form very stable siloxane bonds that, at the same time, can react with hydroxyl groups of wood fibre to form a very strong Si-O bond on the surface of the wood substrate. Siloxane bonds act as coupling agents that improve interfacial bonding between wood dust fibre and recycled PP [36]. Aside from silanol, the reaction of amino, as named amine, interacts instantly with the polymer’s carboxyl groups, producing secondary amide bonds. During processing, secondary amide groups react with the continuously forming carboxyl groups, resulting in imide groups [56]. As a result, for better adhesion between wood and recycled PP, the composite filament containing wood dust with silane treatment has less voids, hence resulting in the lower water absorption percentage, followed by 3/NO at 10%, r-PP at 9%, and 3/NS at 7%.

However, since alkaline treatment increases the availability OH groups in cellulose and hemicellulose, the resulting hydrophilic wood fibres are more able to absorb water via hydrogen bonding between water molecules and the OH-groups on the surface of the wood fibre [57,58]. However, it is still lower than untreated since untreated does not undergo any treatment that changes or improves the bond between the fibre and the matrix, which might result in voids in the composite [53].

### 3.3. Mechanical Properties of r-WoPPC Filament

**Figure 13:**
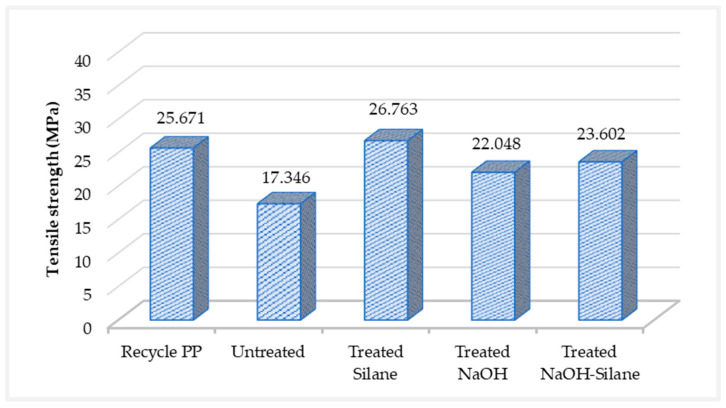
Wire pull test.

The ability of the matrix and fiber to adhere well to one other has a significant impact on the interfacial bonding that determines the composite’s mechanical performance, which results in higher strength. Highest strength can be achieved by a good stress distribution between the matrix and fibre bonding [59]. The results of a wire pull test for r-PP and r-WoPPC filaments treated with NaOH, treated silane, NaOH-silane, and untreated are shown in Figure 13. This experiment should reveal whether the interfacial bonding of the filament is either strong or weak. The highest strength is achieved by good interfacial bonding, whereas the lowest strength is caused by poor interfacial bonding.

The results suggest that silane-treated filaments are stronger compared to all other filaments. The strength of the silane-treated filament improved by 35.2% compared to the untreated filament, confirming that the interfacial adhesion between the recycled PP and the wood fibre has improved. Furthermore, treated silane is compared to r-PP, the result is 4.25% higher, making treated silane stronger than recycled PP as a neat filament. The strength of treated NaOH and NaOH-silane filament is lower than that of silane-treated filament, which is decreased by 17.61% and 11.81%, respectively. This is a similar result as untreated filaments are lower than treated filaments, which is in line with findings by Huang et al. [60]. Moreover, Huang et al. [60] used wood dust fibre reinforced PLA to obtain a low-quality composite, since the wood was not treated and still presented lignin, cellulose, hemicellulose, and pectin.

**Figure 14:**
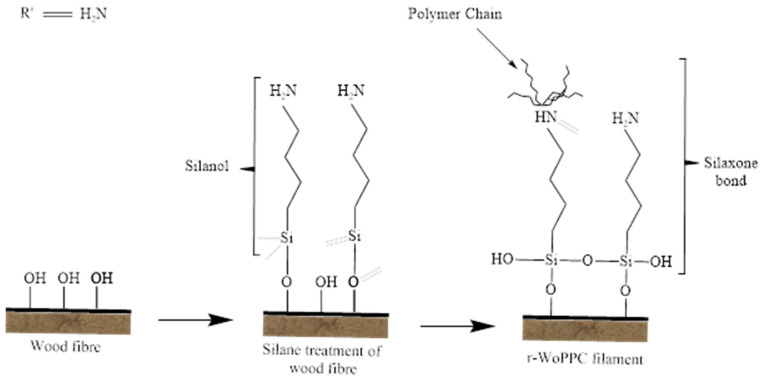
The flow of silane treatment reaction on wood fibre.

**Figure 15:**
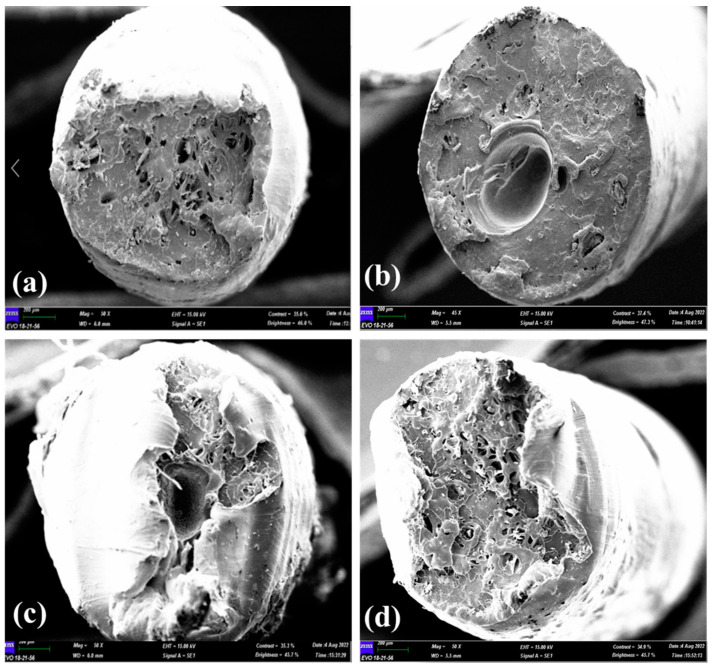
(**a**) 3/SI, (**b**) 3/NS, (**c**) 3/NO, and (**d**).

The results showed that treated silane filament strength is greater than r-PP filament strength. These results indicate that the silane treatment is effective on the wood fibre creating a better bond with the recycled PP. Figure 14 shows the preparation of a silane solution in the presence of water and methanol, where the active substance silanol is formed from aminopropyltriethoxy in silane. Several silanol react with each other to form very stable siloxane bonds, which at the same time, can react with the hydroxyl groups of wood fibre to form very strong bonds on the surface of the wood substrate. These silanol structures underwent a condensation process and naturally deposited on the wood surface [31,36]. This process makes silane a coupling agent that improves interfacial bonding between wood and recycled PP, as shown in Figure 15. The chemical bonding between the r-PP and the silane amino groups incorporated in the fibre surface improved the strength value [47,61]. In addition, the silane treatment effectively removes contaminants such as dust, dirt, and oil on the fibre surface, resulting in stronger interfacial adhesion between r-PP and wood fibers [26], as evidenced by the use of silanes or NaOH-silane mixtures. It has been shown in studies to develop some of the strongest filaments compared to r-WoPPc.

The strength of treated NaOH filament is lower by 14.11% (22.03 MPa) than r-PP at 25.67 MPa, which is similar to fidnings by Ferede et al. [62] when they studied the properties of wood dust/PP composite. They stated that this decreasing strength of NaOH treated fiber composite is possibly due to aggregation of the wood dust or insufficient hydrogen bonding between the r-PP and the wood fibre. The weaker interfacial adhesion between wood and r-PP is also because alkaline treatment removes the amorphous materials in the wood fiber, such as hemicellulose and lignin, which sometimes causes wood surface damage [44,47]. This finding is similar to Ren et al. [53] that developed a composite using kenaf treated with NaOH reinforced into epoxy. Ren Z. et al. (2019) stated that NaOH treatment damaged the cellulose structure in the fibres and degraded the cellulose macromolecular chains and microcrystalline structures. Because cellulose in kenaf fibre is the load-bearing component, its degradation affects kenaf fibre strength.

### 3.4. Morphological Analysis

The fracture surface of the filament wire pull test was examined using SEM. The purpose of this observation was to investigate the adhesion bonding between wood dust fibre and recycled in all the composites filaments. Figure 15 shows the SEM micrographs of the r-WoPPc filament for 3/SI, 3/NS, 3/NO, and 3/UT with 50× magnification.

Among all of the four different filaments, wood fibre reinforced r-PP with silane treatment has the highest wire pull test strength. This highest strength can be related with minimal void and gap that can be seen in the fracture surface micrograph of 3% of wood fibre in Figure 15a. Composite with the least defects is able to withstand a higher load, thus possessing greater strength. The wood dust with other treaments such NaOH-silane, NaOH, and untreated, as shown in the micrograph Figure 15b–d, respectively, has more voids and gaps. Many voids and gaps contributed to weak adhesion between wood fibe and recyled PP, which caused a substantial decrease in the wire pull test strength of the composite [48].

**Figure 16:**
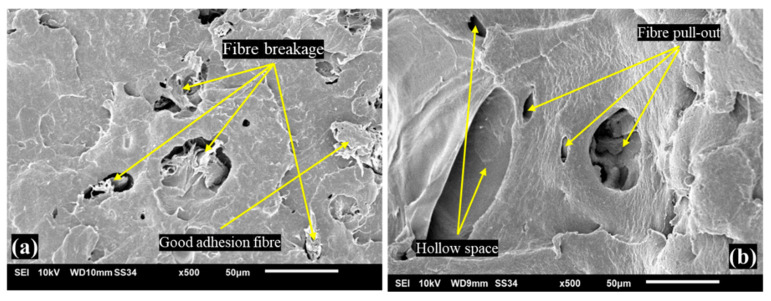
(**a**) Treated silane filament, (**b**) untreated filament.

Figure 16 shows two filament states, treated silane and untreated filaments. According to the observations, treated silane filament shown in Figure 16a has wood fibres remaining on recycled PP after the wire pull test, resulting in fibre breakage, as well as the wood fibres still attached to r-PP, indicating that the fibres treated with silane enhanced the adhesion between fibre and matrix.

Figure 16b illustrates a significant difference where the untreated filament has a hollow space, which could be due to the fibre having detached or not perfectly adhered to the recycled PP. Furthermore, it shows the effect of fibre pull-out test, to which the fibre is extracted during the pull test so that the untreated composite has the lowest strength. According to Aida et al. [36], untreated PP filament is brittle because ti does not undergo treatment; it leaves many a hole on the filament’s surface, when compared to treated and recycled PP filament as a neat filament. As a result, the comparison of treated and untreated filament indicates the weakness of untreated filament, which could not withstand load and was pulled away from the matrix’s grip. This is due to untreated effects that do not perform the diffusion of treatment that can be deployed as a reinforcing agent on polymer filament-reinforced fibre [44].

### 3.5. Fourier Infrared Spectrometry (FTIR)

**Figure 17:**
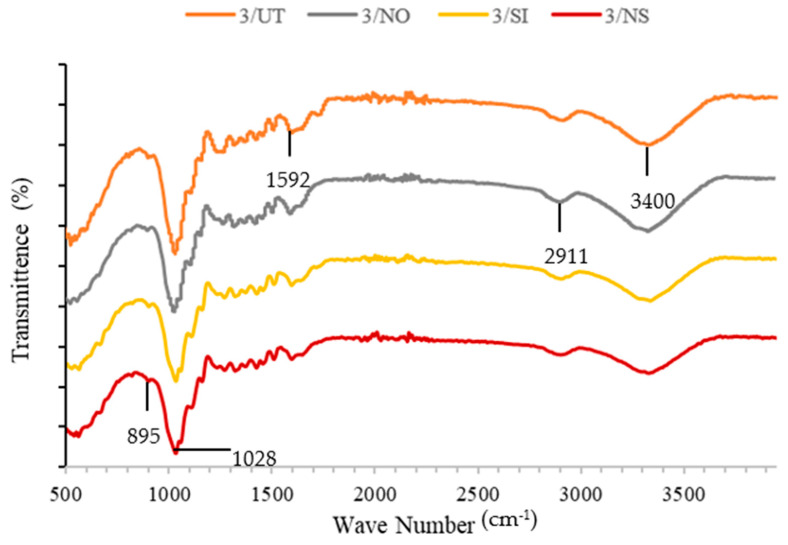
Fourier Infrared Spectrometry (FTIR).

The FTIR spectra of untreated and treated wood dust fibre are shown in Figure 17 and Table 8. The FTIR spectrum showed the presence of cellulose, hemicellulose, and lignin [63]. The group of C-O stretching from lignin, for example in Figure 17, shows a tag at wave 3/NS that was clearly recognised at peaks of 1000–1300 cm^−1^ [40].

Based on the results in Table 8, the 3/UT had a peak of 1030 cm^−1^ for lignin and a peak of 1027 cm^−1^ for 3/NO; the peak for 3/SI and 3/NS fibre was 1028–1029 cm^−1^, respectively. In Table 8 results, the peaks recorded for performing silane treatment appear at 897 and 895 cm^−1^. During silane hydrolysis, NaOH-silane was linked to the formation of silanol groups [44,64]. The peaks recorded that appeared at 1592 cm^−1^ are believed to have emerged from the vibration of the NH_2_ groups in the organo silane agent. It indicated that treatment of silane enhances the wood fibre surface so as to react as a coupling agent where interfacial bonding between wood and recycled PP is improved; similar results were obtained with sisal fibers using aminoethyl amino propyltrimethoxysilane [65,66]. The presence of a peak is recorded in Table 8, which is 1028 cm^−1^ at 3/NS, followed with 1029, 1027, and 1030 cm^−1^ could be identified as the C-O stretching bond of acetyl as a lignin [47,65].

### 3.6. Thermogravimetric Analysis (TGA)

**Figure 18:**
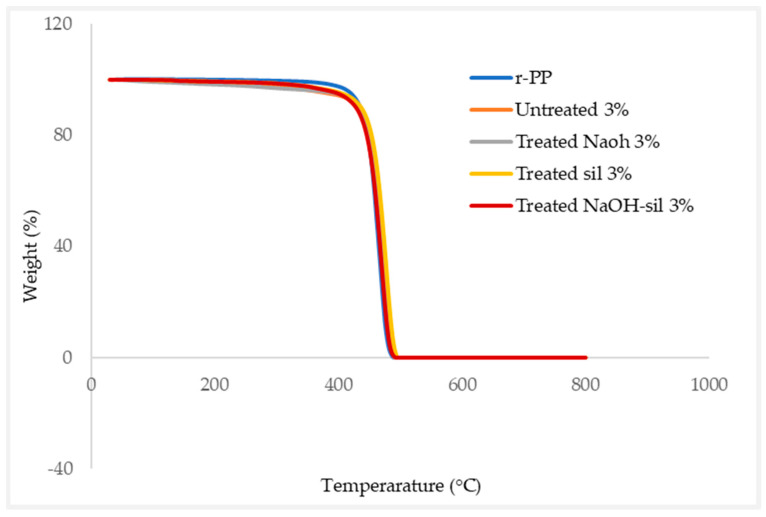
Thermogravimetric analysis in dynamic nitrogen atmosphere.

The TGA analysis of the r-WoPPC filament, which contains treated NaOH, silane, NaOH-silane, and untreated r-PP, is presented in Figure 18. Table 9 summarizes the TGA results of starting dehydration, degradation, decomposition temperature, residue temperature, and final weight after decomposition of all the studied materials.

Referring to Figure 18, it can be observed that the dehydration begins at 35 °C until 140 °C for wood dust treated and untreated dust; the dehydration happened because of the evaporation of moisture content in the wood fibre inside the composite filament. The wood dust composite has a mass loss of not more than 10% during this degradation phase. This finding is similar to Jorge et al. [67], who discovered at an earlier stage of degradation that fibres begin to lose moisture when their moisture evaporates during this phase of usage. Weight loss of fibre at this stage is presently less than 10% of the total weight. Degradation temperature of NaOH-silane filament has a higher temperature compared to untreated and silane treated, possibly because the silane coat fibre and removal of lignin and hemicelluloses during the alkali treatment causes peak temperature compared with the untreated fibre [38,68]. Meanwhile, the temperature range where r-PP degraded was between 10 and 350 ℃. This is the beginning of the evaporation for recycled PP, and the weight loss due to evaporation is now less than 10% [69].

Next, at higher temperatures, between 350–480 °C, the r-WoPPC filament and r-PP begin to decompose when the hemicellulose, cellulose, pectin, and lignin in them are decomposed; similarly, Krishna et al. [3] also reports the chemical composition of the fibre, such as cellulose, hemicellulose, pectin, and lignin, is progressively destroyed at temperatures ranging from 300 to 500 °C. The higher decomposition temperature at 475 °C is r-PP because the sample content is not mixed with other fibres, only recycled PP alone as a neat filament. At this stage, for all samples that have weight left not more than 10%, the same as the literature by Aanimpong et al. [70], are stated as using wood dust reinforced LDPE.

Lastly, at the residue phase of the r-WoPPc composite, higher temperature residue is more than 790 °C; unfortunately, r-PP obtained the lowest temperature at less than 500 °C. The r-WoPPc reduced slightly, whereas the composite’s final thermal decomposition residual ratio increased. The main cause of this condition is the lower thermal decomposition temperature and greater thermal decomposition residual ratio of wood dust [52,71] than that of r-PP. The highest temperature recorded at 803.97 °C on 3/NS was because silane treatment has always been performed; raw wood dust fibres are made up of r-PP surrounded by small amounts of hemicellulose and lignin [72]. The glue between the fibres is made of amorphous structures of hemicellulose and lignin that are softer than wood dust fibre [40]. Other than that, 3/UT decomposed at 794.19 °C, 3/NO decomposed at 798.55 °C, while 3/SI decomposed at 800.19 °C.

The residual weight that might be assigned to char or other products of thermal breakdown [53,73] was less than 1% for every sample of r-WoPPc and r-PP. The TGA result reports that have every silane treatment, such as 3/NS, shows char residue is produced when cellulose is decomposed at a high temperature and that the lowest residual char residue weight indicates better thermal stability, approximate to the findings Aida et al. [36,38].

### 3.7. Differential Scanning Calorimetry (DSC)

**Figure 19:**
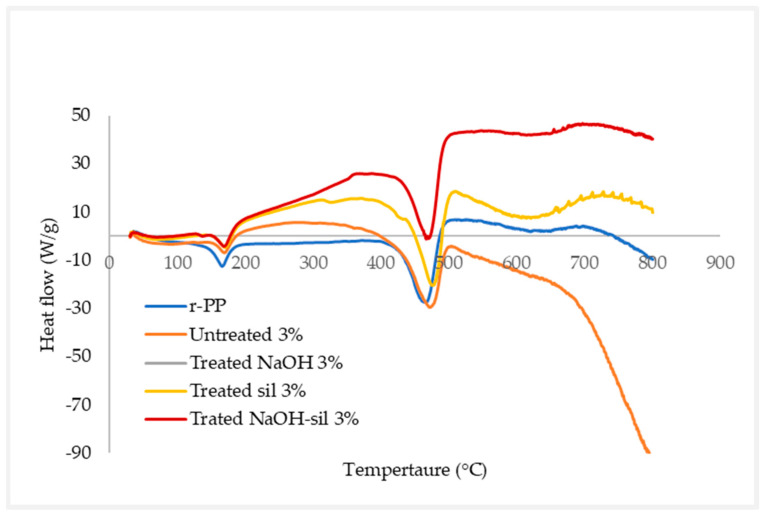
Differential Scanning Calorimetry.

Figure 19 shows the DSC analysis of the r-WoPPC filament, which contains treated NaOH, silane, NaOH-silane, and untreated r-PP Table 10 shows the glass transition (T_g_) and melting temperature (T_m_) data from DSC represented code for 3/UT, 3/NO, 3/SI, 3/NS, and r-PP. To determine if amorphous substances may perform glass transitions, the glass transition (T_g_) temperature and oxidative stability will be studied.

The first peak in Figure 19 represents wood dust fibre, which would be recognized to have an exothermic temperature [54,74]. The r-PP, as a neat polymer filament, setting reference is 167.77 °C, the same as Yubo et al. [52,71], compared to other r-WoPPC, which is 3/UT at 169.22 °C, 3/NO at 171.89 °C, 3/SI at 169.84 °C, and 3/NS at 169.70 °C, respectively, increased with the addition of the wood dust fibre. The glass transition temperature of r-WoPPc is determined by the molecular properties, composition, and compatibility of the composite’s components [55,75]. A component of the melting temperature (T_m_) for r-WoPPc filament and r-PP filament is remarkably different. Using Table 10, the melting temperature may be determined of r-PP at 470.29 °C, 3/UT at 471.13 °C, 3/NO at 470.70 °C, 3/SI at 475.12 °C, and 3/NS at 472.80 °C, respectively. The difference between each data for melting temperature of r-PP and r-WoPPc is in the range of 1–5 °C, indicating that wood dust fibre has a little bit of interfere with the processing temperature, the same as the literature [76]. The highest melting temperature (T_m_) of 475.12 °C on 3/SI found that the crystallization enthalpy and the crystallinity of r-PP can be increased with the addition of wood dust fibre, as compared to Yubo et al. [52,71] obtaining decreased values from using PLA as polymer reinforced. This may be partially caused by the inhibition effect of wood dust fibre on the r-PP crystal formation [57,77]. The number of factors, including the filament’s thermal properties, must be considered before the printing process has started. The reason for this is that filament temperature must be considered as an input parameter throughout the printing process. Insufficient thermal energy throughout the process will influence the quality of the samples and lead to reduced mechanical properties, which is why this is essential to mention.

## 4. Conclusions

In this study, extruder machine pulley speed and nozzle temperature were investigated for the production of wood dust fibres reinforced with recycled polypropylene (r-WoPPc) filaments. The properties of r-WoPPc filaments such as water absorption, mechanical (wire pull strength), morphological, and thermal properties were investigated. The filament was successfully extruded with a machine spool speed of 8 rpm and a nozzle temperature of 180–190 °C. This setting results in a desired thread size of 1.75 mm. The ASTM D792-91 standard is used to determine the water absorption properties of filaments. Filaments with untreated wood absorb a large amount of water (18%) because the wood consists of a lignocellulose material, which absorbs more moisture. Treated wood fibre absorbs less water because the treatment removes the lignocellulose material. Wood dust treated r-WoPPc filament with silane had the highest wire tensile strength compared to all other filaments. The silane strength of treated wood filaments was 35.4% higher than that of untreated wood. The highest strength can be attributed to the minimal void and gap, which can be seen in the SEM micrograph of the fracture surface of the threads. The filament with the least defects, which can withstand a greater load, therefore has a filament with greater strength. The silane treatment increases the interface adhesion between the wood fibre and the recycled PP, the silanol in the silane forms a siloxane bond, which acts as a binder to increase the interface adhesion between the wood and the recycled PP. FTIR analysis confirmed the presence of silanol at a wavelength of 897 cm^−1^ in the treated wood silane filaments. Meanwhile, DSC analysis provides a temperature range of 167–170 °C for the solidification temperature of the extrusion process in the extrusion machine for the production of r-WoPPc filament composites. TGA analysis revealed that wood-treated silane filaments can withstand higher temperatures, especially during the extrusion process, as they have the highest decomposition temperature. In addition, this study shows that the effect of chemical treatment on natural fibres, especially wood dust fibres reinforced with recycled PP, has a significant impact on the yield of several mechanical, physical, thermal, and morphological processes to develop strong bonds for FDM applications in 3D printing to produce components. Examples of this can be found in the automotive or household sector.

## Figures and Tables

**Table 1:** List of mechanical and physical properties of natural fibres [26,27,28].

Fibre	Tensile Strength (MPa)	Young’s Modulus (GPa)	Elongation at Break (%)	Density (g/cm^3^)
Hard Wood	84.9	-	51.75	1.5
Soft Wood	-	11.2	38.5	1.22
Sugar Palm	15.5–290	0.5–3.37	5.7–28.0	1.29
Kenaf	930	53	1.6	1.45
Banana	529–914	27–32	5.9	1.35
Pineapple	413–1627	60–82	14.5	1.44
Coir (coconut husk)	220	6	1.5–2.5	1.25
Bamboo	290	17	-	1.25
Bagasse	350	22	5.8	0.89
Flax Sunflowers	800–1500	60–80	1.2–1.6	1.4

**Table 2:** List of performance treatment utilized with different fibre composite.

Natural Fibre	Polymer Matrix	Findings	Ref.
Wood fibre	Epoxy resin	Alkaline treatment was chosen and obtains better tensile strength and has a better thermal stability test on composites	[35]
Kenaf fibre	PLA	Silane treatment with 2 wt% was used and has tensile strength, and the modulus moves higher compared to untreated fibre composite filament	[36]
Sugar palm fibre	PLA	Employing NaOH-silane treatment results in increased melt flow index and excellent bio composite filament	[37]
Roselle fibre	Vinyl ester	Increased fibre composite tensile strength might well be obtained by silane treatment	[38]
Wood fibre	PLA	Silane treatment was used and resulted in a bio-composite filament with better tensile strength compared to untreated version	[39]
Wood fibre	PP	The 5 wt% silane treatment was used, and the flexural result was greater than the NaOH treatment composite	[40]

**Table 3:** List of composite mixture containing 4 types of treatment.

Filament Composite	Weight of Fibre (g) 3.0%	Weight of Matrix (g) 97.0%	Weight of Composites (g)
Recycled PP (r-PP)	-	-	800
Untreated (3/UT)	24	776	800
NaOH (3/NO)	24	776	800
Silane (3/SI)	24	776	800
NaOH-silane (3/NS)	24	776	800

**Table 4:** Extrusion parameter.

Plastic Type	Barrel Temperature (°C)
Recycled PP	160–200

**Table 5:** Nozzle extrusion temperature.

Nozzle Extrusion Temperature (°C)	Filament Size (mm)
160	2.20–2.60 mm
170	1.80–2.00 mm
180	1.70–1.75 mm
190	1.65–1.75 mm
200	1.55–1.65 mm

**Table 6:** Pulley speed.

Filament Type	Pulley Speed (rpm)	Filament Extrusion Time (s/m)
r-PP	10	6 s/m
3/UT	8.8	13 s/m
3/NO	8.4	15 s/m
3/SI	7.9	18 s/m
3/NS	7.1	23 s/m

**Table 7:** Density of filament.

Sample Code	Density of Filament (g/cm^3^)	Water Absorption Test (%)
r-PP	0.85	9
3/UT	0.82	18
3/NO	0.86	10
3/SI	0.99	8
3/NS	1.01	7

**Table 8:** List FTIR spectra.

	3/UT	3/NO	3/SI	3/NS
Cellulose C^5^H^8^O^4^	3330	3334	3336	3331
Absorption of H2O C=O	1592	1591	1592	1593
Hydroxyl Group -OH Si-C Stretching Bond	3400–3200	3400–3200	3400–3200	3400–3200
-Si-O-Si- Asymmetric Stretching	1030	1027	1029	1028
Hemicellulose CH	2914	2911	2901	2901
Silane (silanol group) -Si-OH	-	-	897	895

**Table 9:** Result of TGA test r-PP and r-WoPPc.

Materials Code	Degradation Temperature (°C)	Decomposition Temperature (°C)	Residue Temperature (°C)	Final Weight after Decomposition (%)
r-PP	330.16	475.93	499.52	0.03
3/UT	36.98	472.77	794.19	0.63
3/NO	71.20	471.37	798.55	0.98
3/SI	36.76	471.63	800.19	0.53
3/NS	136.17	467.64	803.97	0.22

**Table 10:** Results of DSC.

Materials	Glass Transition Temperature, T_g_ (°C)	Melting Temperature, T_m_ (°C)
r-PP	167.77	470.29
3/UT	169.22	471.13
3/NO	171.89	470.70
3/SI	169.84	475.12
3/NS	169.70	472.80
